# GATA1 induces epithelial-mesenchymal transition in breast cancer cells through PAK5 oncogenic signaling

**DOI:** 10.18632/oncotarget.2999

**Published:** 2015-01-21

**Authors:** Yang Li, Qiang Ke, Yangguang Shao, Ge Zhu, Yanshu Li, Nanxi Geng, Feng Jin, Feng Li

**Affiliations:** ^1^ Department of Cell Biology, Key Laboratory of Cell Biology, Ministry of Public Health, and Key Laboratory of Medical Cell Biology, Ministry of Education, China Medical University, Shenyang, China; ^2^ Department of Breast Surgery, Department of Surgical Oncology, Research Unit of General Surgery, The First Affiliated Hospital of China Medical University, Shenyang, China

**Keywords:** EMT, Breast cancer, GATA1, PAK5, Phosphorylation

## Abstract

Epithelial-mesenchymal transition (EMT) is a key process in tumor metastatic cascade that is characterized by the loss of cell-cell junctions, resulting in the acquisition of migratory and invasive properties. E-cadherin is a major component of intercellular junctions and the reduction or loss of its expression is a hallmark of EMT. Transcription factor GATA1 has a critical anti-apoptotic role in breast cancer, but its function for metastasis has not been investigated. Here, we found that GATA1, as a novel E-cadherin repressor, promotes EMT in breast cancer cells. GATA1 binds to *E-cadherin* promoter, down-regulates E-cadherin expression, disrupts intercellular junction and promotes metastasis of breast cancer cell *in vivo*. Moreover, GATA1 is a new substrate of p21-activated kinase 5 (PAK5), which is phosphorylated on serine 161 and 187 (S161 and S187). GATA1 recruits HDAC3/4 to *E-cadherin* promoter, which is reduced by GATA1 S161A S187A mutant. These data indicate that phosphorylated GATA1 recruits more HDAC3/4 to promote transcriptional repression of *E-cadherin*, leading to the EMT of breast cancer cells. Our findings provide insights into the novel function of GATA1, contributing to a better understanding of the EMT, indicating that GATA1 and its phosphorylation may play an important role in the metastasis of breast cancer.

## INTRODUCTION

Occurrence of metastasis due to tumor progression is the primary cause of most cancer-related deaths. For carcinomas, which originate from epithelial cells and account for most tumors, acquisition of invasiveness and motility requires them to undergo a dramatic transition to a mesenchymal state (epithelial-mesenchymal transition or EMT) [1, 2]. In the process of EMT, cancer cells disrupt their intercellular junctions and employ developmental processes to gain migratory and invasive properties [3, 4]. Dissemination of tumor cells from the primary lesion is the most common event in the metastatic process and leads to the shedding of millions of carcinoma cells into the circulation each day [5].

GATA1, the founding member of the GATA transcription factor family, binds the core consensus DNA sequence (T/A) GATA (A/G) and is central to the differentiation, proliferation, and/or apoptosis of erythroid [6] and megakaryocytes cells [7]. GATA1 regulates many genes expression depending upon its ability to bind both DNA and various different protein partners [8]. In breast cancer aggressiveness, GATA1 is identified as a key feature by enhancing survivin expression [9]. It was reported that Prx5 protected cells from oxidative stress-mediated apoptosis in a GATA1-regulated manner [10]. However, the function of GATA1 in breast cancer metastasis remains unclear.

The p21-activated kinases (PAKs), a conserved family of serine/threonine kinases, function as dynamic signaling nodes in cancer and are central to a variety of cellular functions [11–13]. The six isoforms of PAKs in human are classified into group I (PAK1–3) and group II (PAK4–6) [14]. PAK5 plays a critical role in promoting cytoskeletal reorganization, including filopodia formation and neurite outgrowth [15]. In addition, Xenopus active PAK5 decreases cell adhesion when expressed in hemisphere and inhibits the calcium-dependent reassociation of cells [16]. Recently, PAK5 has been shown to promote cells metastasis by regulating cell adhesion and migration [17]. Interestingly, in response to various external stimuli, PAK5 shuttles from mitochondria to the nucleus [18], indicating that it may play a role in regulating gene transcription, although it is unknown.

In this study, we found GATA1 functions as a novel E-cadherin repressor that binds to *E-cadherin* promoter and represses the *E-cadherin* transcription. In addition, GATA1 is a new physiological substrate of PAK5, which is phosphorylated on serine 161 and 187. Further, GATA1 wild type but not GATA1 S161A S187A mutant promoted breast cancer cell invasion *in vitro* and metastasis *in vivo*. Meanwhile, we showed that phosphorylated GATA1 by PAK5 recruits more HDAC3/4 to promote transcriptional repression of *E-cadherin*, leading to the EMT of breast cancer cells. These data indicate that GATA1 and its phosphorylation may play a critical role in cancer progression.

## RESULTS

### GATA1 binds to *E-cadherin* promoter and down-regulates E-cadherin

**Figure 1:**
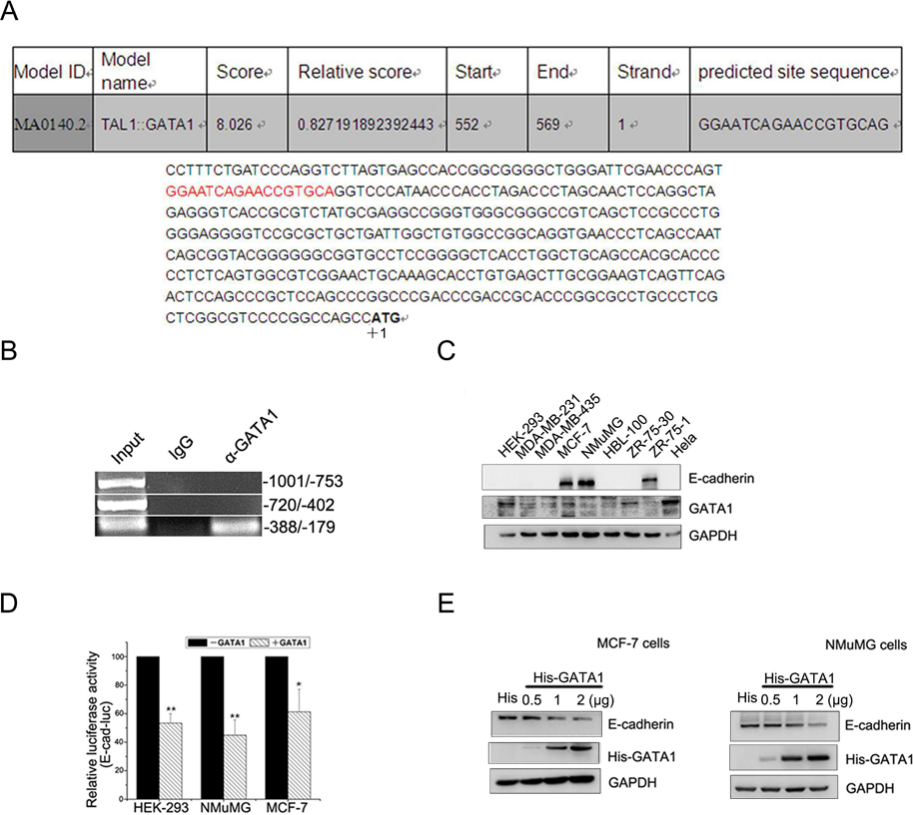
GATA1 binds to *E-cadherin* promoter and down-regulates E-cadherin **(A)** Nucleotide sequence of the *E-cadherin* promoter was analyzed. Potential transcription factor binding motifs are red. ATG is indicated by +1. **(B)** GATA1 binds to *E-cadherin* promoter (–388/–179) detected by ChIP assays. **(C)** Protein expression levels of E-cadherin and GATA1 in mammary cell lines. **(D)** HEK-293, NMuMG and MCF-7 cell lines were transfected with pGL2-E-cad-luc, pRL-TK and pcDNA-GATA1 or control plasmid for luciferase assays. *p < 0.05, **p < 0.01. **(E)** MCF-7 and NMuMG cells were transfected with 0.5 μg, 1 μg, 2 μg His tagged-GATA1 plasmid, and western blot analysis was performed.

It has been reported that GATA1 is overexpressed in aggressive breast cancer [9] and GATA3, another GATA family member, inhibits breast cancer metastasis through increasing E-cadherin expression [19]. As we know, down-regulation of E-cadherin is associated with the development of invasive carcinoma, metastatic dissemination and poor prognosis [20, 21]. To identify the *cis*-elements involved in the function of GATA1 on *E-cadherin* transcription, the sequence within the proximal promoter region of the human *E-cadherin* gene was analyzed (Figure 1A) [22]. The result revealed one GATA1 binding site located at –349/–332 upstream of ATG. Also, ChIP assay result showed that GATA1 bound to *E-cadherin* promoter at –388 to –179, which contained the motif (Figure 1B, lower lane). We further identified the expression of GATA1 and E-cadherin in different mammary cell lines. The results showed that GATA1 was in high expression while E-cadherin was lost in ZR-75-30 cells. Meanwhile, GATA1 was in low expression and E-cadherin in high expression in NMuMG, MCF-7 and ZR-75-1 cells (Figure 1C). These data indicate a negative relationship between the expression of GATA1 and E-cadherin in some breast cancer cell lines. Thus we speculated that GATA1 might regulate E-cadherin expression. To confirm the down-regulation of *E-cadherin* by GATA1, we carried out luciferase assays in HEK-293, NMuMG and MCF-7 cell lines. The result showed that GATA1 did down-regulate *E-cadherin* promoter activity in these three cell lines to a different degree (Figure 1D). Furthermore, the protein level of E-cadherin decreased with the increasing amounts of transfected his-tagged GATA1 in MCF-7 cells and NMuMG cells (Figure 1E). These data demonstrate that GATA1 represses E-cadherin expression.

### GATA1 recruits HDAC3/4 to down-regulate *E-cadherin* transcription

**Figure 2:**
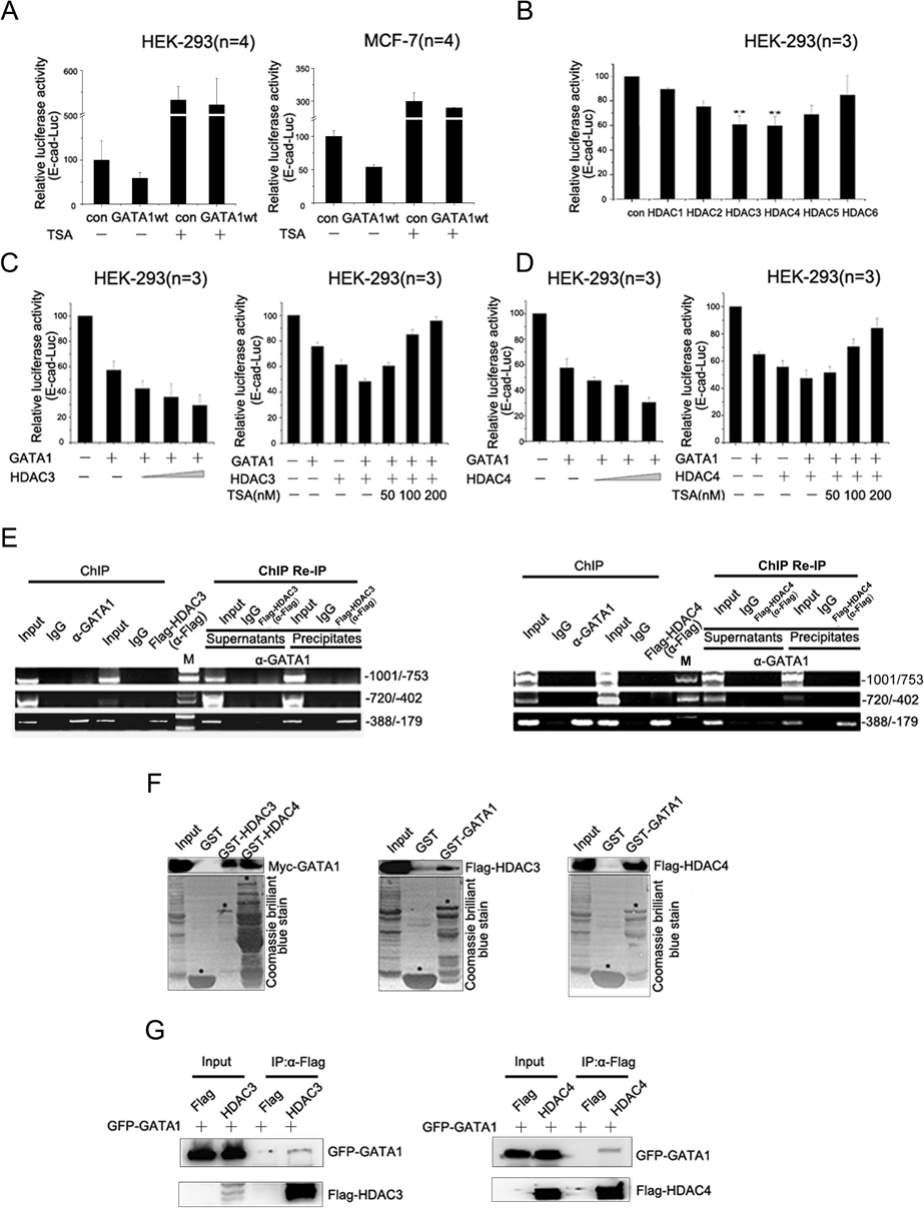
GATA1 recruits HDAC3/4 to down-regulate *E-cadherin* transcription **(A)** pGL2-E-cad-luc and pRL-TK plasmids were co-transfected with pcDNA-GATA1 or control plasmid into HEK-293 cells and MCF7 cells. Then cells treated with or without TSA for luciferase assay. **(B)** HEK-293 cells were transfected with pGL2-E-cad-luc plasmid together with HDAC constructs expressing HDAC1–6, respectively. **p < 0.01. **(C–D)** HEK-293 cells were transfected with pGL2-E-cad-luc, pcDNA-GATA1 and increasing amounts of HDAC3/4 as indicated for Luciferase Assays. Simultaneously, increasing amounts of TSA was added to HEK-293 cells transfected with GATA1 and HDAC3/4 for Luciferase Assays. **(E)** MCF-7 cells were transfected with Flag-HDAC3/4 expression plasmids. ChIP assay was carried out using anti-GATA1 or anti-Flag antibody, followed by PCR with primers amplifying the *E-cadherin* promoter region (–1001/–753, –720/–402, –388/–179). ChIP Re-IP, soluble chromatin, prepared from MCF-7 cells transfected with Flag-HDAC3/4, was firstly immunoprecipitated with antibody against GATA1, then reimmunoprecipitated with anti-Flag antibody. **(F)** *In vitro* translated Myc-GATA1 or Flag-HDAC3/4 was incubated with GST-HDAC3/4 or GST-GATA1 fusion proteins for GST pull-down assay. **(G)** HEK-293 cells were transfected with GFP-GATA1 and Flag-HDAC3 or GFP-GATA1 and Flag-HDAC4. Lysates were immunoprecipitated with Flag antibodies and immunoblotted with Flag and GFP antibodies.

Histone deacetylation is one of the best-characterized covalent modifications associated with gene transcriptional repression [23], so we wonder if GATA1 recruits HDACs to down-regulate *E-cadherin* transcription. The luciferase assays showed that inhibition of HDACs activity by TSA, a known HDACs inhibitor, resulted in the elevation of *E-cadherin* promoter activity (Figure 2A). Thus, GATA1 down-regulated *E-cadherin* promoter activity through histone deacetylation. We further tested the effect of six HDACs (HDAC1–6) on *E-cadherin* transcriptional regulation by GATA1. The luciferase assay results showed that the six HDACs exerted distinct repressive effect on *E-cadherin* promoter activity, among which HDAC3/4 had a much more prominent effect on *E-cadherin* repression (Figure 2B). Moreover, HDAC3/4 enhanced the inhibitory effect of GATA1 on *E-cadherin* promoter activity in a dose-dependent manner and this effect could be dose-dependently reversed by TSA (Figure 2C–2D). Next, the ChIP assay showed that HDAC3/4 bound the same region (–388/–179) of the *E-cadherin* promoter as GATA1 and the ChIP Re-IP assay indicated that HDAC3/4 and GATA1 acted in a combinatorial fashion on the *E-cadherin* promoter (Figure 2E). To test whether GATA1 could physically interact with HDAC3/4, *in vitro* GST-pull down assays were performed and the results indicated that GATA1 bound to HDAC3/4 directly (Figure 2F). In addition, co-immunoprecipitation assays confirmed the interaction of GATA1 with HDAC3/4 *in vivo* (Figure 2G). Taken together, these results indicate that GATA1 recruits HDAC3/4 to down-regulate E-cadherin expression.

### GATA1 is a physiological substrate of p21-activated kinase 5

**Figure 3:**
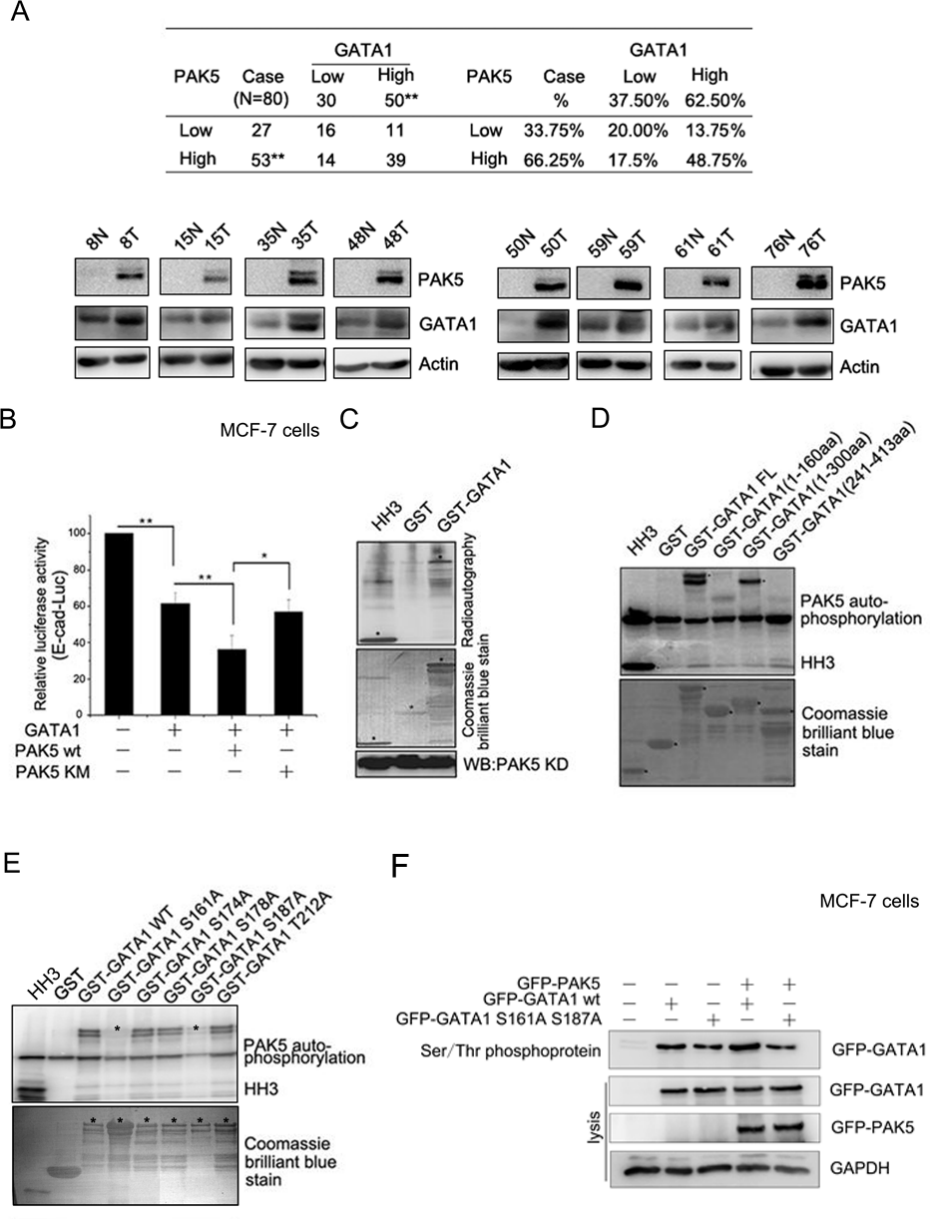
GATA1 is a physiological substrate of p21-activated kinase 5 **(A)** Western blot analysis demonstrates the protein level of PAK5 and GATA1 in breast cancer tissues and matched adjacent noncancerous tissues. N, matched adjacent noncancerous tissue; T, breast cancer tissue. **p < 0.01. **(B)** PAK5wt/KM and GATA1 were transfected into MCF-7 cells as indicated for Luciferase Assays. *p < 0.05, **p < 0.01. **(C)** HEK-293 cells transfected with Myc-PAK5KD (PAK5 kinase domain) were lysed for IP with anti-Myc antibody, the immunoprecipitated PAK5 kinase was incubated with GST or GST-GATA1 for *in vitro* kinase assay. Histone H3 (HH3) served as a positive control. **(D)** *In vitro* kinase assay using commercial PAK5 kinase and GST-GATA1FL (Full length, 1–413aa) or GST-GATA1 deletion mutants (1–160aa, 1–300aa and 241–413aa). Histone H3 (HH3) served as a positive control. **(E)** *In vitro* kinase assay using commercial PAK5 kinase and GST-GATA1 wt or GST-GATA1 single-site mutations as indicated. **(F)** MCF-7 cells transfected with GATA1 wt/S161A S187A and PAK5 wt were used for Ser/Thr phosphoprotein purification. Then concentrated protein was used for western blot. The top lane, the phosphorylated GATA1 from these cells lysates was used for immunoblotting using anti-GFP antibody. Total cells lysates were used for immunoblotting by anti-GFP, GAPDH antibodies.

It is reported that PAK5 regulates cell adhesion and migration in colorectal carcinoma cells [17]. PAK5 was in higher expression in breast cancer tissues than matched adjacent noncancerous tissues (Figure 3A), which was consistent with the previous report [24]. The high expression rate of the PAK5 and GATA1 were 66.25% (53/80) and 62.50% (50/80) in breast cancer tissues and low expression rate 33.75% (27/80) and 37.50% (30/80) in matched adjacent noncancerous tissues (Figure 3A). A significant statistical difference was found between the two groups. Importantly, the high level of PAK5 expression correlated with the expression of GATA1 in breast cancer tissues (*p* = 0.033). Since E-cadherin is one of the most important cell-cell adhesion proteins and many kinases are required for the disruption of E-cadherin-based cell-cell junctions, such as PAK1 and CDKL2 [25, 26], we presumed that PAK5 might modulate E-cadherin expression. To test whether the phosphorylation of GATA1 by PAK5 was related to GATA1's inhibitory effect on *E-cadherin*, luciferase assays were performed. As expected, it was wild type PAK5 (PAK5 wt) rather than kinase dead PAK5 (PAK5 KM) promoted transcriptional repression of *E-cadherin* by GATA1 (Figure 3B). So, we wondered if GATA1 was a physiological substrate of PAK5. The *in vitro* kinase assay showed that PAK5 phosphorylated GST-GATA1 (Figure 3C). To identify specific PAK5 phosphorylation sites in GATA1, first, we used GST-GATA1 FL (1–413aa) and GST-GATA1 deletion constructs (1–160aa, 1–300aa and 241–413aa) for *in vitro* kinase assay. The data indicated that the phosphorylation sites were located between amino acids 160 and 240 (Figure 3D). Next, we created single-site mutations of the predicted sites in GATA1 amino acids 160–240 (S161A, S174A, S178A, S187A, T212A) for kinase assay. As shown in Figure 3E, both mutations of Ser161 to Ala and Ser187 to Ala significantly impaired PAK5 phosphorylation of GATA1. Then, Serine/Threonine phosphoprotein purification kit was used to further test the PAK5-mediated GATA1 phosphorylation *in vivo* (Figure 3F). Total Ser/Thr phosphorylated protein from cell lysates were analyzed by western blot. The results showed that phosphorylated wild type GATA1 but not GATA1 mutant was increased with overexpressing PAK5 (Figure 3F, the top lane, compare lane 4 with lane 2 and compare lane 5 with lane 3). Since *in vitro* kinase assay showed Ser161 was main phosphorylation site, phosphor-GATA1 Ser161 antibody was used to further test the PAK5-mediated GATA1 phosphorylation in cells (Figure S1). These results indicate that both GATA1 Ser161 and Ser187 may be the main phosphorylation sites by PAK5.

### PAK5-mediated GATA1 phosphorylation regulates EMT in breast cancer cells

**Figure 4:**
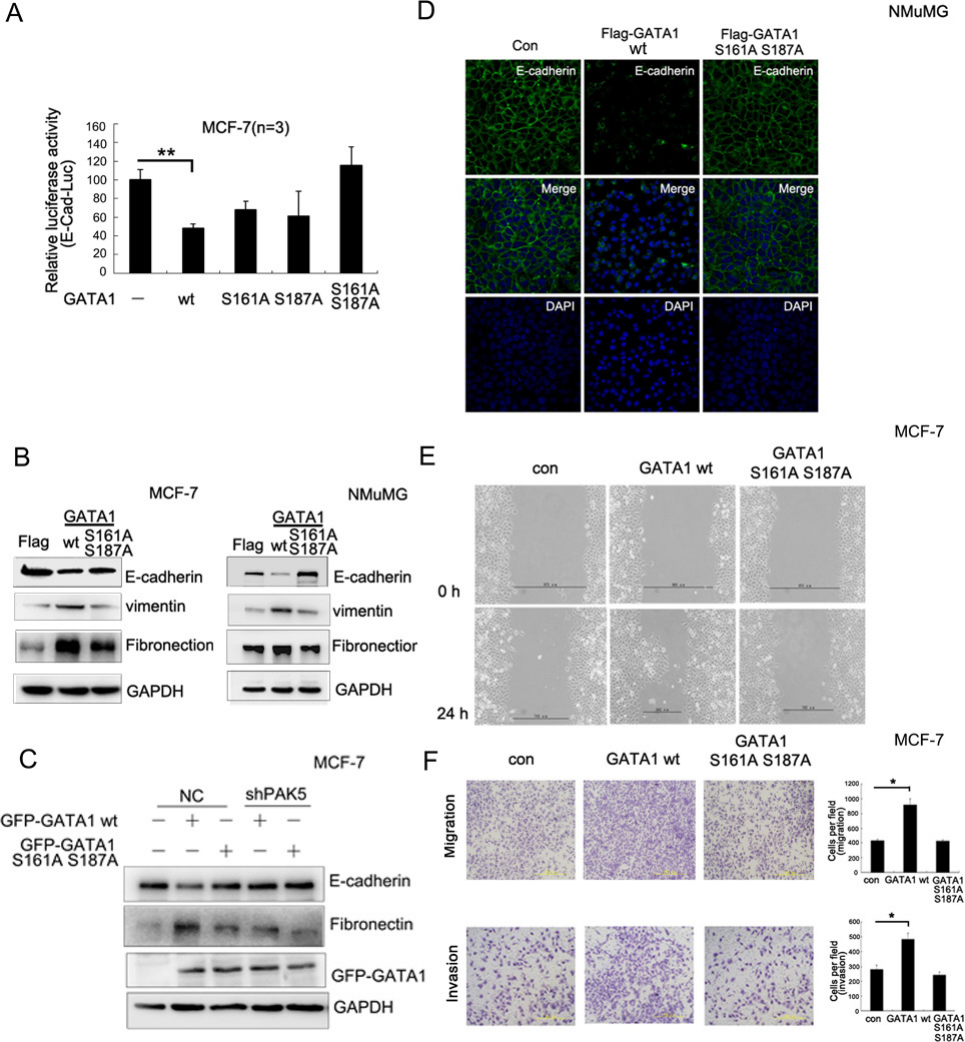
PAK5-mediated GATA1 phosphorylation regulates EMT in breast cancer cells **(A)** MCF-7 cells were transfected with wild type or different mutants of GATA1 for Luciferase assay. **p < 0.01. **(B)** Empty vector, Flag-GATA1 wt or Flag-GATA1 S161A S187A was stably expressed in MCF-7 and NMuMG cells through lentivirus. Cell lysates from these cells were used for immunoblotting using anti-Fibronectin, E-cadherin, vimentin, GAPDH antibodies. **(C)** Stably knocked down shPAK5 or NC (non-specific control) cells were transfected with GATA1 wt. Western blot analyzed the marker of EMT. **(D)** The same transfection as (B), NMuMG cells were cultured 24 hours and analyzed by immunofluorescence using anti-E-cadherin antibody followed by Alexa Flour 488 (green) antibody and nucleus was stained by DAPI (blue). **(E–F)** Effects of different types of GATA1 over-expression on the migration of cultured MCF-7 cells were examined by wound-healing (E) and transwell migration chambers (F) assays. Results are representative of three independent experiments. Migrated cells were plotted as the average number of cells per field of view. In the low lane, transwell migration chambers were treated with 10% matrigel, but not in the top lane. *p < 0.05.

To further analyze whether GATA1-induced EMT depended on the phosphorylation by PAK5, MCF-7 cells were transfected with different mutants of GATA1. The results showed that only GATA1 S161A or S187A partially reversed *E-cadherin* promoter activity, whereas GATA1 S161A S187A mutant completely reversed *E-cadherin* promoter activity (Figure 4A), suggesting that there was a collaboration between GATA1 serine 161 and 187. Furthermore, stable infection with GATA1 wt, but not GATA1 S161A S187A mutant resulted in decreased E-cadherin expression and increased fibronectin, vimentin expression in MCF-7 cells or NMuMG cells (Figure 4B). To demonstrate the importance of PAK5 in GATA1 phosphorylation at both Ser161 and 187, endogenous PAK5 in MCF-7 cells was knocked down by two different siRNAs (2# and 3#). SiRNA 1# is a control for off target effects of the siRNA (Figure S2A). Then MCF-7 cells were stably infected with shPAK5 (3#), and the efficiency of PAK5 knockdown was also observed (Figure S2B). To further analyze the functional role of PAK5 in GATA1-mediated EMT, PAK5 knockdown MCF-7 cells were transfected with GATA1 wt or GATA1 mutant. As shown that PAK5 knockdown suppressed the EMT of MCF-7 cells induced by GATA1 wt (Figure 4C, compare lane 4 with lane 2). The results suggest that PAK5 may play an important role in promoting EMT induced by GATA1. The EMT-related mesenchymal conversion were characterized by immunofluorescence. As shown in Figure 4D, when NMuMG cells infected with GATA1 wt, the expression of E-cadherin in cell membrane were significantly decreased (Figure 4D). To evaluate the roles of GATA1 wild type and mutant in cell migration, we performed both wound-healing and transwell migration assays. Up-regulation of GATA1 wt strongly facilitated directional cell migration toward a “wound” in a cell monolayer (Figure 4E), but not GATA1 S161A S187A. Up-regulation of GATA1 wt also markedly enhanced cell migration and invasion through a permeable filter (Figure 4F). These observations suggest that EMT induced by GATA1 is dependent on the phosphorylation of GATA1 by PAK5 at both Ser161 and Ser 187 sites. Therefore, GATA1 may interrupt intercellular junction, enforce mesenchymal phenotypes and promote cell migration through the PAK5-mediated phosphorylation of GATA1.

### Phosphorylation of GATA1 enhances breast cancer cell metastasis *in vivo*

**Figure 5:**
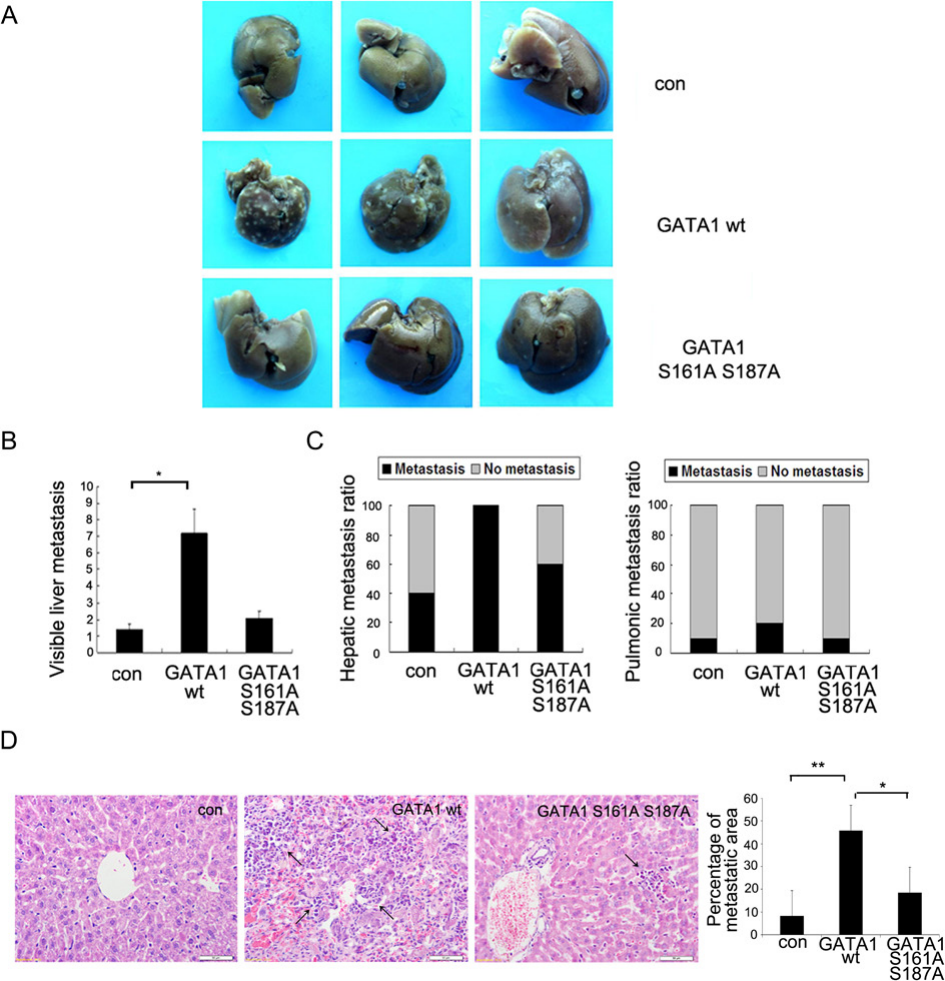
Phosphorylation of GATA1 enhances breast cancer cell metastasis *in vivo* 1 × 10^7^ MCF-7 cells stably expressing empty vector, GATA1 wt or GATA1 S161A S187A were injected into nude mice through tail vein. **(A)** Livers were dissected after injection and macroscopically photographed. **(B)** Graphical representation of the number of liver metastases from each mouse (mean ± s.d.); *p < 0.05. **(C)** Effect of MCF7 cells with stable expression of control Flag, Flag GATA1 wt, Flag GATA1 S161A S187A on incidence of liver and lung metastasis. **(D)** Representative image of H&E-stained liver sections of mice and percentage of metastatic liver surface area relative to tatal live surface area. scale bar, 50 μm. 30 independent visions were counted. *p < 0.05, **p < 0.01.

To monitor the effects of GATA1 on tumor cell metastasis *in vivo*, MCF-7 cells infected with GATA1 wt, GATA1 S161A S187A or control vector were injected into mice tail vein. Eight weeks after injection, multiple tumor nodules were observed in the livers of GATA1 wt mice (Figure 5A–5B). Pathological analysis of tumor metastasis revealed that only four of ten control mice developed into a liver metastatic phenotype, and one of ten mice exhibited lung metastasis (Figure 5C). However, after receiving GATA1 wt cells, all ten mice developed distant tumors in the liver, and only two of these mice developed lung metastasis (Figure 5C). Meanwhile, we found the tumor malignancy of GATA1 S161A S187A cells was similar to control cells, only six of ten GATA1 S161A S187A mice underwent a liver metastatic phenotype, and one of ten mice exhibited lung metastasis (Figure 5C). Hematoxylin and eosin staining confirmed that GATA1 wt expression resulted in much more markedly metastatic spread to the livers of the mice compared to GATA1 S161A S187A and control vector (Figure 5D). The results showed that liver metastasis was more significant than lung metastasis. These findings indicate PAK5-mediated GATA1 phosphorylation promotes breast cancer metastasis *in vivo*.

### The phosphorylated GATA1 recruits more HDAC3/4 to promote transcriptional repression of E-cadherin

**Figure 6:**
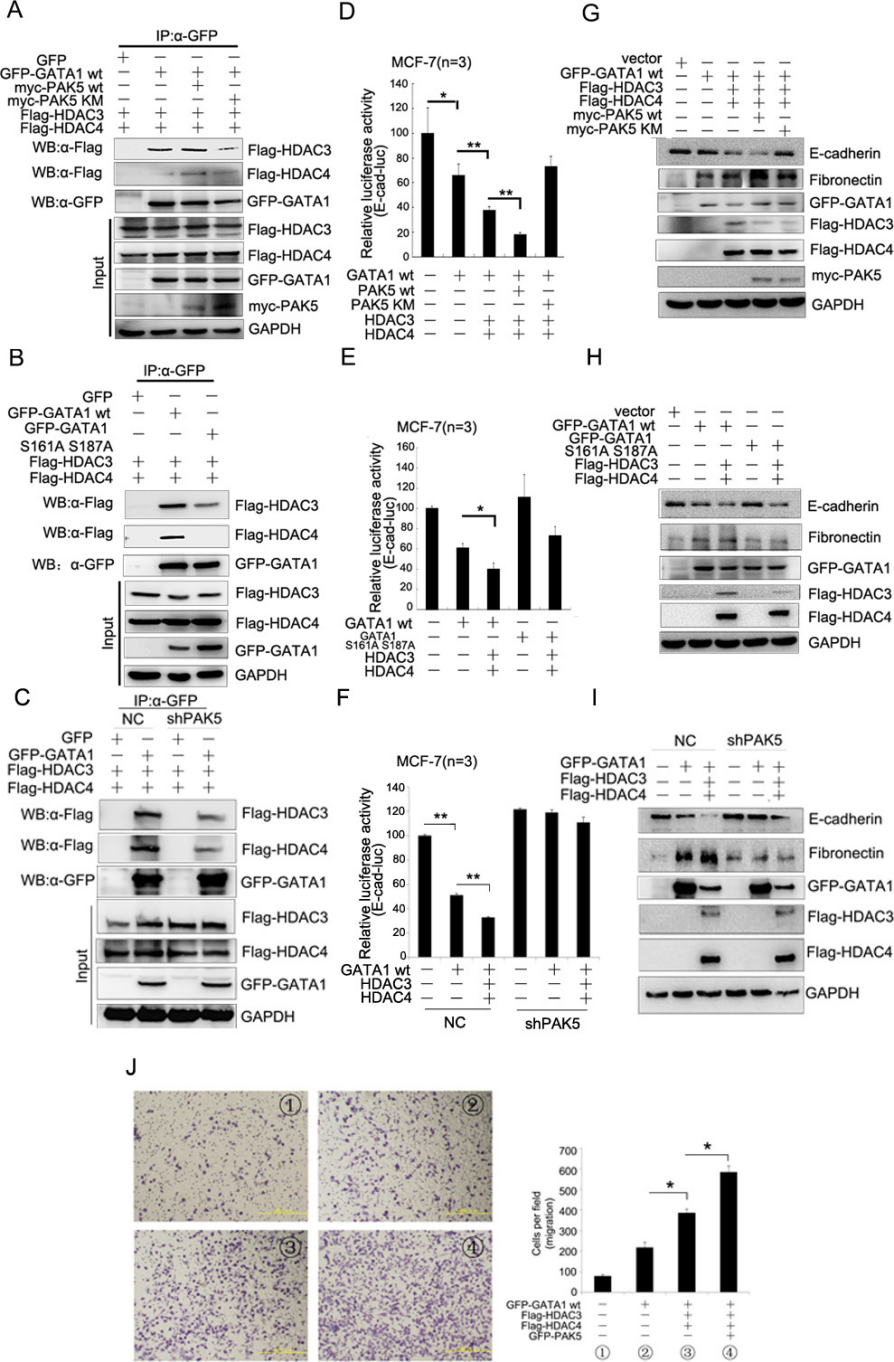
The phosphorylated GATA1 recruits more HDAC3/4 to promote transcriptional repression of E-cadherin **(A)** HEK-293 cells were transfected with GFP-GATA1, Myc-PAK5wt/KM, Flag-HDAC3/4 as indicated. Lysates were immunoprecipitated with anti-GFP antibody and immunoblotted with anti-Flag, anti-GFP and anti-myc antibody. **(B)** HEK-293 cells were co-transfected with different combination of plasmids as indicated. Lysates were immunoprecipitated with anti-GFP antibody and immunoblotted with anti-Flag and anti-GFP antibody. **(C)** MCF-7 cells stably infected with shPAK5 were co-transfected with different combination of plasmids as indicated. Lysates were immunoprecipitated with anti-GFP antibody and immunoblotted with anti-Flag and anti-GFP antibody. **(D)** PAK5wt/KM and GATA1, HDAC3/4 were transfected into MCF-7 cells as indicated for Luciferase Assays. *p < 0.05, **p < 0.01. **(E)** GATA1 wt and GATA1 S161A S187A, HDAC3/4 were transfected into MCF-7 cells as indicated for Luciferase Assays. *p < 0.05. **(F)** GATA1, HDAC3/4 were transfected into stably PAK5 knockdown MCF-7 cells as indicated for Luciferase Assays. **p < 0.01. **(G–I)** The same transfection as D, E, F cell lysates from these cells were used for immunoblotting using anti-Fibronectin, E-cadherin antibodies. **(J)** GATA1, HDAC3/4 and PAK5 were transfected into MCF-7 cells, then transwell assay was used. Results are representative of three independent experiments. Migrated cells were plotted as the average number of cells per field of view. *p < 0.05.

To examine whether PAK5-mediated GATA1 phosphorylation affected recruitment of HDAC3/4, co-immunoprecipitation assay was performed and the result showed that transfection with PAK5 wt, but not PAK5 KM in HEK-293 cells, increased the association of GATA1 with HDAC3/4 (Figure 6A). As seen in Figure 6B, GATA1 S161A S187A mutant markedly reduced the association of GATA1 with HDAC3/4, especially HDAC4. In addition, PAK5 knockdown also markedly reduced the association of GATA1 with HDAC3/4 (Figure 6C). All the results above indicated that the association of GATA1 with HDAC3/4 was dependent on the GATA1 phosphorylation by PAK5. Then, luciferase assays were carried out to determine the effect of phosphorylated GATA1 and HDAC3/4 on *E-cadherin* transcription. As expected, it was PAK5 wt rather than PAK5 KM promoted transcriptional repression of *E-cadherin* by GATA1-HDAC3/4 complex (Figure 6D and Figure S3A). Overexpressed GATA1 wt but not GATA1 S161A S187A mutant in combination with HDAC3/4 markedly inhibited *E-cadherin* promoter activity in MCF-7 and HEK-293 cells (Figure 6E and Figure S3B). However, when stably infected with shPAK5, MCF-7 cells did not show a reduction in *E-cadherin* promoter activity even in the overexpression of GATA1 and HDAC3/4 (Figure 6F). Since all these proteins regulated *E-cadherin* transcription, we wondered if they could affect E-cadherin mRNA level. Then, qRT-PCR assay showed each protein down-regulated the E-cadherin mRNA level (Figure S3C). Meanwhile, we found that these proteins had a coordination role in repression of E-cadherin mRNA level. Finally, to detect whether the phosphorylated GATA1-HDAC3/4 complex was important to the acquisition of EMT, western blot analysis showed that PAK5 wt rather than PAK5 KM obviously inhibited E-cadherin expression and promoted fibronectin expression by GATA1-HDAC3/4 complex (Figure 6G). Overexpressed GATA1 wt but not GATA1 S161A S187A mutant in combination with HDAC3/4 markedly inhibited the E-cadherin expression (Figure 6H). Knockdown of PAK5 inhibited EMT mediated by GATA1-HDAC3/4 complex in breast cancer cells (Figure 6I, compare lane 5 with lane 2 or compare lane 6 with lane 3). In the process of EMT, cells gain migratory property. In Figure 6J, we found that HDAC3/4 and PAK5 markedly enhanced GATA1-mediated cell migration. Based on these results, we propose that the phosphorylated GATA1 by PAK5 recruits more HDAC3/4 to the *E-cadherin* promoter, thus promotes transcriptional repression of *E-cadherin*, leading to the EMT of breast cancer cells.

## DISCUSSION

EMT is a process vital for morphogenesis during embryonic development and has also been implicated in the transition of early-stage tumors into invasive malignancies. Till now, there are several transcription factors which have been demonstrated to be capable of inducing EMT. These include repressors which bind to *E-cadherin* promoter directly, such as Snail, Slug, ZEB1, ZEB2, SIP1, E47 and Twist [27–31]. Here we have shown a new repressor of E-cadherin–GATA1. GATA3, another GATA family member, inhibits breast cancer metastasis through increasing E-cadherin expression [19]. Interestingly, we found GATA1 had the opposite function. It was reported that GATA1 was overexpressed in breast cancer tissues [9] and we found that there was a negative relationship between the expression of GATA1 and E-cadherin in NMuMG, MCF-7, ZR-75-1 and ZR-75-30 cell lines. We also found that GATA1 could inhibit the promoter activity of *E-cadherin*, the protein expression of E-cadherin. Since down-regulation of E-cadherin expression plays an essential role in tumor aggressiveness, these data indicate that GATA1 may play a critical role in breast cancer metastasis, although further data should be provided.

Here we have shown a new repressor of E-cadherin–GATA1. GATA3, another GATA family member, was found to induce E-cadherin expression through binding GATA-like motifs located at –182 and –214 bp in the *E-cadherin* promoter [19]. These data indicate that GATA1 and GATA3 exert distinct effect on *E-cadherin* transcriptional regulation by binding different motifs in the *E-cadherin* promoter. In addition, we have evidence that GATA1 disrupts cell-cell junction and promotes the migration of breast cancer cells. It is recently reported that GATA1 is overexpressed in breast carcinomas [9]. Thus, we speculate that GATA1 might be involved in EMT and the metastasis of breast cancer.

Histone deacetylation is a major epigenetic modification that contributes to repressing gene expression, and HDACs are the effectors of histone deacetylation [23]. Here, we have demonstrated that GATA1 recruits HDAC3/4 to repress *E-cadherin* transcription. It is well-known that transcription factor phosphorylation is closely related to gene transcriptional regulation [32]. It has been reported that Akt directly phosphorylated GATA1 at serine 310 [33], and PAK5 was in higher expression in breast cancer tissues than matched adjacent noncancerous tissues. As a serine/threonine protein kinase, PAK5 may phosphorylate GATA1 and promote the repression of E-cadherin by GATA1. As expected, our results demonstrated that GATA1 was a novel substrate for PAK5 kinase and PAK5 but not other members of PAK family was correlated with the repression of E-cadherin by GATA1. In addition, we defined GATA1 Ser161, Ser187 were the main phosphorylation sites by PAK5. To further analyze whether GATA1-induced EMT depended on its phosphorylation by PAK5, MCF7 cells were infected with different kinds of GATA1, we found GATA1wt might impair intercellular junction, enforce mesenchymal phenotypes and promote cell migration through the PAK5-mediated phosphorylation of GATA1. Our result, therefore, indicate that phosphor-Ser161/Ser187 is a novel regulatory mechanism by which GATA1 promotes breast cancer cell migration and invasion. The fact that inhibition of Ser161/187 phosphorylation suppressed metastasis in mouse xenografts raises the possibility of inhibiting Ser161 Ser187 phosphorylation in prevention of breast cancer metastasis.

Recent studies have revealed that ATM/ATR-mediated phosphorylation of DBC1 promotes binding to SirT1 [34]. Phosphorylation of RUNX1 by CDK reduces direct interaction with HDAC1 and HDAC3 [35]. We wonder the relationship between GATA1 phosphorylation and histone deacetylation in *E-cadherin* transcription. Here, we demonstrate that the phosphorylated GATA1 by PAK5 recruits more HDAC3/4 to promote transcriptional repression of *E-cadherin*. Our results couple PAK5-mediated GATA1 phosphorylation to histone deacetylation in *E-cadherin* transcription regulation.

**Figure 7:**
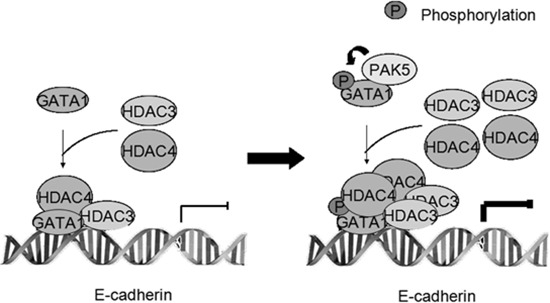
Proposed model showing the role of GATA1 and its phosphorylation in repressing E-cadherin transcription

Based on the previous knowledge, as well as findings from this study, we propose a hypothetic model to illustrate the role of GATA1 and its phosphorylation in repressing *E-cadherin* transcription (Figure 7). PAK5 phosphorylates the transcription factor GATA1 mainly at Ser161 and Ser 187, phosphorylated GATA1 recruits more HDAC3/4 to the promoter of *E-cadherin* and consequently suppresses the transcription of *E-cadherin* gene and promotes the EMT of breast cancer cells (Figure 7).

In conclusion, we uncovered a novel function of GATA1 in regulating EMT, identified GATA1 as a novel repressor of E-cadherin and a new substrate for PAK5. We coupled GATA1 phosphorylation to histone deacetylation in *E-cadherin* transcription regulation in breast cancer cells. Our findings provide insights into the new function of GATA1, and could lead to a better understanding of the *E-cadherin* transcription regulation. Our data indicate that GATA1 and its phosphorylation may play a critical role in the metastasis of breast cancer, which might open up new potential therapeutic avenues for the treatment of breast cancer.

## MATERIALS AND METHODS

### Plasmid constructs

The human PAK5WT/K478M expression plasmids were gifts from Dr. Jonathan Chernoff (Cotteret and Chernoff, 2006; Cotteret et al., 2003) and the HA-hGATA1 plasmid was a gift from Dr. Trang Hoang (Lahlil et al., 2004). GFP- and Myc-tagged PAK5 or GATA1 and GST-tagged GATA1 were constructed by PCR and subcloned into pEGFP-C1 (ClonTech), pcDNA3.1/Myc-HisA (Invitrogen) and pGEX-5X-1 (GE Healthcare) vectors respectively. Plasmids expressing human HDAC1–6 (fused to the Flag-epitope) were gifts from Dr. E. Seto, we subcloned hHDAC3/4 cDNA into pGEX-5X-1 vector by *Eco*RI to obtain GST-tagged HDAC3/4.

### Cell culture, transfections and luciferase dual reporter assays

Human embryonic kidney HEK-293 cell line, normal mouse mammary epithelial cell line NMuMG and human breast cancer cell lines MDA-MB-231, MDA-MB-435, HBL-100, MCF-7, ZR-75-1 and ZR-75-30 were cultured in DMEM (Dulbecco's modified Eagle's medium) containing 10% FBS (fetal bovine serum). HEK-293, NMuMG, MCF-7 cell lines were cotransfected with the listed constructs according to the manufacturer's instructions by using Lipofectamine 2000 (Invitrogen). After 24 h of transfections, cells were harvested, washed, and lysed in 100μl of passive lysis buffer (Promega). Luciferase activities were analyzed using a Promega dual-luciferase reporter assay system. Firefly luciferase activity was normalized to the activity of Renilla luciferase control. Relative luciferase activity was analyzed using the luminometer Lumat LB 9507 (Berthold Technologies, Germany). All the results represent the means ± SD based on at least three independent experiments.

### Immunoprecipitation, western blot analysis and GST pull-down assays

For immunoprecipitation, the cells were washed by cold PBS twice before being lysed in IP lysis buffer supplemented with protease and phosphatase inhibitors. Total protein lysate (2 mg) was used for each immunoprecipitation using specific antibody, Protein A agarose beads (GE Healthcare Uppsala, Sweden) were added to the cells and incubated overnight 4°C. Washed precipitated proteins were analyzed by western blot. The immunoprecipitation (IP), western blot and GST pull-down assays used in this study have been described previously in detail [23]. The samples were incubated with anti-E-cadherin (BD), anti-PAK5 (R&D), anti-GATA1/-Myc (Santa Cruz), anti-pGATA1 S161 (Bioss), anti-GFP (Genscript), anti-fibronectin (Sigma), anti-Flag (Shang Hai Ruixing), anti-His (Genscript), anti-GAPDH (Kang Chen, as a loading control) antibodies.

### Immunofuorescence

Cells were fixed in methanol at room temperature for 20 min and then blocked with normal goat serum for 30 min. The cells were incubated with the primary antibody overnight at 4°C and subsequently with secondary antibody conjugated with Alexa Fluor 488 (Green) dye from Molecular Probes after washed three times in PBT (PBS with 1‰ triton x-100). The DNA dye DAPI (Molecular Probes) was used (blue). Confocal scanning analysis was performed by using a Leika laser confocal scanning microscope in accordance with established methods, utilizing sequential laser excitation to minimize the possibility of fluorescent emission bleed-through.

### ChIP (chromatin immunoprecipitation) and ChIP Re-IP

Transfected MCF-7 cells were cross-linked with 1% formaldehyde (final concentration) after washing. Cells were lysed with lysis buffer and sonicated on ice, then precleared with protein A-agarose. Following immunoprecipitation with anti-GATA1 (Santa Cruz) or anti-Flag (Sigma) antibodies, protein complexes were immunoprecipitated and washed in turn with low salt, high salt, lithium chloride buffer and TE buffer. After elution and reverse cross-linking, the purified DNA was resuspended in TE buffer. DNA samples (2 μl) were then amplified by PCR. Primer pairs for the *E-cadherin* promoter were: E-cad (–1001/–753) sense: 5′-CGTCTGTACTATAAATACATAAT-3′, antisense: 5′-TAGGATTTCAGGTGTGAGCCAT-3′. E-cad (–720/–402) sense: 5′-TCAGACTAGGAGATCGAGACCAGGC-3′, antisense: 5′-AAGGGCTTTTACAATTGGCTGAGTT-3′. E-cad (–388/–179) sense: 5′-CTTAGTGAGTCACCGGCGGGGCTGG-3′, antisense: 5′-CCGCTGATTGGCTGAGTGTTCACCT-3′. For ChIP Re-IP and ChIP Re-IPs assays were undertaken using a previously describe method [23].

### Transwell migration assay

To assess cell migration *in vitro*, MCF-7 cells (1 × 10^5^ cells in 100 μl DMEM supplemented with 1% FBS) were placed in the top chamber of transwell migration chambers (8 μm BioCoat Control Inserts, Becton Dickinson Labware, Bedford, MA). The lower chamber was filled with 600 μl DMEM supplemented with 10% FBS. After 24 h, unmigrated cells were removed from the upper surface of the transwell membrane with a cotton swab, and migrated cells on the lower membrane surface were fixed, stained, photographed, and counted under high-power magnification.

### PAK5 kinase assay

GST-fusion proteins were purified *in vitro* and washed three times with kinase buffer (50 mM HEPES, pH 7.5, 10 mM MgCl_2_, 2 mM MnCl_2_ and 0.2 mM DTT). Commercialized PAK5 Kinase (Cell Signaling Technology Inc.) or immunoprecipitated cell synthesized PAK5 kinase domain was used for kinase assay in 50 μl of kinase buffer added with 10 μCi of [γ-^32^P] ATP (5,000 Ci/mmol) and 2.5 μM cold ATP for 30 min at 30°C. Reactions were stopped by addition of 6 × SDS loading buffer. After 10% SDS-PAGE and transferred onto PVDF membranes, ^32^P-labelled proteins were visualized by autoradiography with Molecular Imager RX (BIO-RAD). Histone H3 (Invirogen) was used as positive control. Ponceau stain indicated the loading amounts of GST-fusion proteins.

### Ser/Thr phosphoprotein purification assay

The Ser/Thr phosphoprotein was purified, according to the manufacturer's instructions by using PhosphoProtein Purification Kit (Qiagen No. 37101). Take a volume of lysate containing 2.5 mg of total protein, and adjust the protein concentration to 0.1 mg/ml. Finally, 30 μl concentrating protein was used for western blot.

### Lentiviral infection

PAK5-shRNA and control lentivirus were obtained from Shanghai Genechem Co., Ltd. The PAK5-shRNA target sequence was 5′-CAAAGTCTTCGTACCTGAATC-3′. Virus supernatant was incubated on target cells for 24 hours with 8 μg/ml polybrene, following the manufacturer's instructions. Infected cells were selected in puromycin, as optimized for cell line.

### *In vivo* metastasis assay

MCF-7 cells with stable expression of control Flag, Flag-GATA1 wt and Flag-GATA1 S161A S187A were trypsinized washed once with PBS and resuspended in PBS (1 × 10^7^ cells/ml). Two hundred microliters of cell solution were injected into the tail vein of 5- to 6-week-old female BALB/c mice (Vital River Laboratory Animal Technology Co. Ltd., Beijing, China). Eight weeks after injection, the mice were killed with anesthesia. The lungs and livers were extracted and fixed in 4% paraformaldehyde in phosphate-buffered saline. Liver tissues were embedded in paraffin, sectioned and stained with hematoxylin and eosin. Visible liver metastases were measured and counted using a microscope. All experimental procedures involving animals were conducted in accordance with the Guide for the Care and Use of Laboratory Animals (NIH publication no. 80–23, revised 1996) and were performed according to the institutional ethical guidelines for animal experiments.

### RNA isolation and qRT-PCR

Total RNA was isolated using Trizol reagent (Invitrogen, Carlsbad, USA). Total RNA (2 μg) was used for the synthesis of first-strand cDNA using M-MLV reverse transcriptase (Invitrogen, Beijing, China). Quantitative real-time PCR was performed using the SYBR green mix (Applied Biosystems). The reactions were performed with a 7500 Fast Real-Time PCR System (Applied Biosystems). Sequences of the RT-PCR primers were as follows (5′–3′): E-cadherin (GCCTCCTGAAAAGAGAGTGGAAG and TGGCAGTGTCTCCAAATCCG) GAPDH (ACCACAGTCCATGCCATCAC and TCCACCACCCTGTTGCTGTA).

## SUPPLEMENTARY FIGURES


