# Supplementary material for: GATA1 induces epithelial-mesenchymal transition in breast cancer cells through PAK5 oncogenic signaling
Source: Oncotarget. 2015 Jan 21;6(6):4345–56. doi: 10.18632/oncotarget.2999 (PMC4414194; doi:10.18632/oncotarget.2999)
Supplement: Supplementary file 1 [file oncotarget-06-4345-s001.pdf]

SUPPLEMENTARY FIGURES

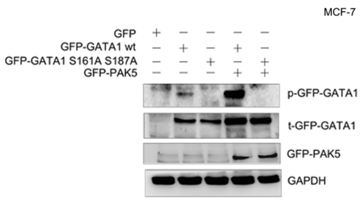

Supplementary Figure S1: PAK5 phosphorylates GATA1 *in vivo*. MCF-7 cells transfected with GATA1 wt/S161A S187A and PAK5 wt were used for western blot with phosphor-GATA1 Ser161 antibody.

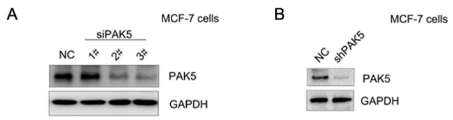

Supplementary Figure S2: MCF-7 cells were stably tranduced with lentiviral shPAK5. (A) MCF-7 cells were transfected with three different siPAK5 or the non-targeting control siRNA (NC) for western blot. **(B)** MCF-7 cells were stably tranduced with lentiviral shPAK5 or the non-targeting control shRNA (NC) for western blot.

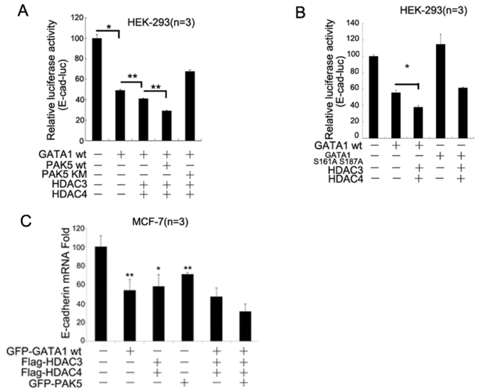

Supplementary Figure S3: PAK5-HDAC3/4-GATA1 have a coordination role in E-cadherin down-regulation. (A) PAK5wt/KM and GATA1, HDAC3/4 were transfected into HEK-293 cells as indicated for Luciferase Assays. *$p < 0.05$, **$p < 0.01$. **(B)** GATA1 wt and GATA1 S161A S187A, HDAC3/4 were transfected into HEK-293 cells as indicated for Luciferase Assays. *$p < 0.05$. **(C)** MCF-7 cells were transfected with GATA1, PAK5, HDAC3/4 or all the plasmids, then qRT-PCR was used. Results are representative of three independent experiments. *$p < 0.05$, **$p < 0.01$.
